# RPA-primed droplet-partitioned CRISPR/Cas12a assay for low-copy detection of *Candida albicans* in vaginal specimens

**DOI:** 10.3389/fcimb.2026.1960866

**Published:** 2026-10-28

**Authors:** Ju Long, Juan Xie, Fang Liu, Jie Peng, Wen-Fei Xu, Xu-Hui Li

**Affiliations:** 1 College of Fisheries and Life Science, Shanghai Ocean University, Shanghai, China; 2 Zhejiang Key Laboratory of Multiomics and Molecular Enzymology, Yangtze Delta Region Institute of Tsinghua University, Zhejiang, Jiaxing, China; 3 Second Affiliated Hospital of Jiaxing University, Jiaxing, China; 4 Women’s Hospital, School of Medicine, Zhejiang University, Hangzhou, China

**Keywords:** *Candida albicans*, CRISPR/Cas12a, droplet-partitioned detection, recombinase polymerase amplification, vulvovaginal candidiasis

## Abstract

*Candida albicans* is a major cause of vulvovaginal candidiasis, and sensitive detection of low-abundance fungal DNA remains challenging in clinical specimens. In this study, we developed a two-stage assay combining rapid recombinase polymerase amplification (RPA) with droplet-partitioned CRISPR/Cas12a fluorescence detection. Target DNA was first amplified by RPA for 10 min and subsequently analyzed in vortex-generated polydisperse droplets without microfluidic chip fabrication. Using predefined NTC-based analytical criteria, the operational LoDs were 10 copies/μL for the bulk RPA-Cas12a assay and 1 copy/μL for the droplet-partitioned format under the tested conditions. No cross-reactivity was observed with the tested non-target organisms, including additional clinically relevant non-*albicans Candida* species. In a preliminary evaluation of 28 residual vaginal swab specimens, the assay showed complete concordance with a routine composite clinical reference using an exploratory cohort-derived droplet-count cutoff. Because the comparator was not species-specific for *C. albicans*, these findings represent preliminary concordance rather than definitive diagnostic accuracy. The complete workflow was performed within approximately 1 h using basic laboratory equipment. These findings demonstrate that droplet partitioning can improve the low-copy analytical readout of RPA-Cas12a detection and provide a practical approach for qualitative identification of *C. albicans DNA* in vaginal specimens.

## 1 Introduction

Vulvovaginal candidiasis (VVC) is a common fungal infection that affects a large proportion of women worldwide, with *Candida albicans* (*C. albicans*) being the predominant causative pathogen. Typical manifestations include vulvar pruritus, erythema, swelling, irritation, and abnormal vaginal discharge, and recurrent or persistent infection can substantially impair quality of life (Yano et al., 2019; Nyirjesy et al., 2022). Therefore, rapid and accurate detection of *C. albicans* can provide useful microbiological information in the evaluation of patients with suspected VVC. However, detection of *C. albicans* alone does not establish active VVC because asymptomatic vaginal colonization can occur.

Current laboratory approaches for diagnosing Candida-associated vaginitis mainly include microscopy, fungal culture, and nucleic acid-based tests (Nyirjesy et al., 2022). Although culture-based methods are widely used in clinical laboratories, they are relatively time-consuming and may not be optimal for rapid decision-making, particularly when fungal burden is low. Accordingly, there is a continued need for molecular diagnostic strategies that combine high sensitivity, short turnaround time, and operational simplicity.

CRISPR/Cas12a-based nucleic acid detection has emerged as a powerful diagnostic platform because of its programmable target recognition and trans-cleavage activity toward single-stranded DNA reporters (Chen et al., 2018; Li et al., 2018). Upon binding to the target sequence, the crRNA-guided Cas12a complex is activated and cleaves nearby reporter molecules, thereby producing a fluorescence signal (Chen et al., 2018). However, conventional bulk CRISPR/Cas12a assays often show limited sensitivity at low target concentrations, and many reported digital CRISPR platforms rely on monodisperse microfluidic droplets and specialized instrumentation, which restrict their broader use (Park et al., 2021; Yue et al., 2021).

Recombinase polymerase amplification (RPA) is an isothermal amplification technology with several attractive features, including mild reaction temperature, short amplification time, and minimal equipment requirement (Lobato and O'Sullivan, 2018). Integration of RPA with CRISPR/Cas12a can substantially improve analytical sensitivity (Ji et al., 2025). Meanwhile, partitioning the reaction into droplets can spatially confine Cas12a activation events, suppress background accumulation, and improve signal discrimination. These features make RPA-assisted droplet CRISPR readouts attractive for rapid pathogen detection (Xu et al., 2025; Zeng et al., 2025).

RPA-assisted CRISPR/Cas12a detection offers rapid target amplification under isothermal conditions, but the final fluorescence signal is commonly measured in a bulk reaction. At low target concentrations, bulk fluorescence may remain difficult to distinguish from background because target-triggered reporter cleavage and basal signal accumulation occur within the same reaction volume. Reaction partitioning provides a potential means of spatially confining Cas12a activation events and improving discrimination of low-abundance targets. However, the analytical contribution of droplet partitioning after RPA amplification requires direct evaluation against the corresponding bulk RPA-Cas12a format.

In this study, we developed a two-stage RPA-Cas12a assay followed by vortex-assisted polydisperse droplet partitioning for *Candida albicans* DNA detection. The study was designed to determine whether: (i) rapid RPA could provide sufficient target amplification within 10 min; (ii) droplet partitioning could improve low-copy detection relative to the corresponding bulk fluorescence assay; and (iii) the resulting workflow could classify vaginal clinical specimens using an exploratory cohort-derived positive-droplet cutoff. We systematically optimized the RPA and Cas12a reaction conditions, screened crRNAs in the amplified-target context, compared the analytical performance of bulk and droplet formats, and conducted a preliminary clinical evaluation using vaginal secretion specimens.

## 2 Materials and methods

### 2.1 Reagents and materials

The recombinant plasmid containing the target sequence and all primers were synthesized by Sangon Biotech (Shanghai, China). The ssDNA-FQ reporter and Cas12a crRNAs were synthesized by Bio-Life Science Co., Ltd. (Guangzhou, China). LbaCas12a and NEBuffer r2.1 were purchased from New England Biolabs. ddH_2_O and NaCl were obtained from Sangon Biotech. Bovine serum albumin (BSA) and isopropyl palmitate were purchased from Macklin (Shanghai, China). The TwistAmp Basic Kit was obtained from TwistDx (Cambridge, UK). Genomic DNA from *Candida albicans*, *Pseudomonas aeruginosa*, *Staphylococcus aureus*, *Acinetobacter baumannii*, *Escherichia coli*, and *Candida glabrata* was purchased commercially. A yeast genomic DNA extraction kit was purchased from Tiangen Biotech (Beijing, China). All oligonucleotide sequences used in this study are listed in **Supplementary Table S1**.

### 2.2 Target sequence, primer, and crRNA design

The reference sequence used for assay design was derived from the ITS1–5.8S–ITS2 region of *Candida albicans* (GenBank accession no. OW987719.1). The 198-bp RPA amplicon spans part of the ITS1 region and the adjacent 5.8S rRNA gene. The forward primer F1 and the crRNA2 protospacer are located within ITS1, whereas the reverse primer R4 is located within the 5.8S rRNA gene. The crRNA2 protospacer is located within ITS1 and is adjacent to a TTTC PAM. The sequences of the RPA primers, crRNAs, and fluorescence reporter used in this study are provided in **Supplementary Table S1**.

For interspecies sequence comparison, homologous ITS-region sequences from *C. auris, C. dubliniensis, C. parapsilosis, C. tropicalis*, and *C. krusei* were retrieved from NCBI and aligned using DNAMAN version 9. For assessment of intraspecies conservation, ITS-region sequences from 11 *C. albicans* clinical isolates were retrieved from NCBI and aligned using the same software. The isolate designations and corresponding NCBI accession numbers are listed in **Supplementary Table S2**.

### 2.3 RPA reaction

Unless otherwise specified, the RPA reaction described below corresponds to the final conditions used in subsequent experiments. The RPA reaction was carried out in a total volume of 25 μL containing rehydration buffer, lyophilized enzyme pellet, forward and reverse primers, MgOAc, and 2.5 μL of template DNA. For analytical sensitivity experiments, the reported target concentrations refer to the concentration of the template before addition to the RPA reaction. Therefore, template concentrations of 10 and 1 copy/μL corresponded to nominal inputs of 25 and 2.5 copies per 25 μL RPA reaction, respectively. Based on the optimization results described in Section 2.5 and the Results section, the final RPA incubation used for subsequent experiments was performed at 37°C for 10 min. For agarose-gel verification only, an aliquot of the RPA product was purified by phenol/chloroform/isoamyl alcohol extraction before electrophoresis. RPA products used for downstream Cas12a detection were transferred directly without this purification step.

### 2.4 Bulk CRISPR/Cas12a fluorescence assay

Unless otherwise specified, the bulk CRISPR/Cas12a fluorescence assay described below represents the final working conditions used in subsequent experiments. The bulk CRISPR/Cas12a reaction was performed in a total volume of 12.5 μL containing 300 nM LbaCas12a, 450 nM crRNA, 500 nM ssDNA-FQ reporter, 1× NEBufferTM r2.1, and the indicated amount of target DNA. For RPA-Cas12a detection, 1.25 μL of RPA product was added to each 12.5 μL CRISPR/Cas12a reaction. The amplified product was not quantitatively measured after RPA; therefore, target concentrations reported in the analytical sensitivity experiments refer to the pre-RPA template concentrations. The reaction mixture was briefly centrifuged and incubated at 41°C for 60 min in the ABI Q3 system, and fluorescence was recorded every 1 min. As described in Section 2.5, reaction temperature, Mg^2+^ concentration, BSA concentration, Cas12a concentration, and the crRNA: Cas12a ratio were systematically screened.

### 2.5 Optimization of the RPA and Cas12a systems

To optimize the RPA reaction, the effects of temperature (33, 35, 37, and 39°C), primer concentration (0–600 nM), and Mg^2+^ concentration (0–30 mM) were investigated by agarose gel electrophoresis. Based on the comparative results and the subsequent amplification kinetics analysis, 37°C was selected as the working temperature for downstream experiments, and the optimized reaction reached effective amplification within approximately 10 min.

To optimize Cas12a activity, real-time fluorescence assays were conducted at different temperatures (35, 37, 39, 41, 43, 45, and 47°C). Mg^2+^ concentration, BSA concentration, Cas12a concentration, and crRNA: Cas12a ratios were also evaluated. The overall optimization outcome was summarized in the heatmap shown in the Results section, and the final bulk and droplet assays thereafter followed the working reaction conditions reported in Sections 2.4 and 2.6.

### 2.6 PddRPACas assay

The PddRPACas workflow was conducted using the final RPA and Cas12a working conditions described above unless otherwise indicated. The assay consisted of two steps. First, target DNA was pre-amplified by RPA. Second, 1.25 μL of RPA product was added as the final component to the 12.5 μL CRISPR/Cas12a reaction mixture, and this addition was defined as the start of the detection reaction. Immediately after mixing, a 3 μL aliquot of the reaction mixture was transferred into 100 μL of predispensed oil containing 90% isopropyl palmitate and 10% ABIL EM 180 (v/v). Emulsification was initiated within approximately 1 min after addition of the RPA product and was performed by vortexing at 2500 rpm for 5 s to generate polydisperse droplets. The resulting emulsions were immediately incubated at 41 °C for 45 min. After incubation, 20 μL of each emulsion was transferred onto a glass slide using a cut pipette tip, and fluorescence imaging was initiated within 5 min using the Nikon fluorescence microscope. The same timing sequence was applied to all technical replicate reactions.

To minimize RPA amplicon carry-over contamination, all experimental workflows were physically separated by dedicated independent rooms. Nucleic acid extraction of clinical specimens was conducted within a biosafety cabinet. Reagent preparation, RPA amplification, post-amplification tube opening and transfer into CRISPR-Cas12a reaction mixture, and fluorescent signal imaging were performed in separate dedicated rooms. A unidirectional workflow was maintained; amplified products were transferred only from the amplification area to the designated post-amplification area and were never returned to reagent-preparation or nucleic-acid-extraction areas. Non-template controls (NTCs) were incorporated in each experimental run to monitor potential amplicon contamination.

### 2.7 Specificity evaluation

Genomic DNA from *C. albicans* was used as the target. The non-target panel included *P. aeruginosa, S. aureus, A. baumannii, E. coli, C. glabrata, C. tropicalis, C. parapsilosis, C. krusei, C. auris* and *C. dubliniensis*. Reference sequence accession numbers for the seven *Candida* species included in the analytical specificity evaluation are provided in **Supplementary Table S3**. Non-target DNA was tested at 100 copies/μL under the same assay conditions, and ddH_2_O was included as an NTC. Three separately prepared technical replicate reactions were analyzed for each organism. No organism was considered cross-reactive unless its reaction-level signal exceeded the predefined analytical positivity threshold.

### 2.8 Extracted vaginal DNA matrix-interference assessment

To evaluate potential interference from vaginal specimen-derived components remaining after DNA extraction, a matrix-interference experiment was performed using genomic DNA extracted from one vaginal swab specimen that was negative according to the composite clinical reference and independently confirmed negative for *C. albicans* by qPCR. Three target levels were evaluated using *C. albicans* genomic DNA at pre-RPA concentrations of 20, 100, and 500 copies/μL, representing low-, intermediate-, and high-positive levels, respectively. Each RPA reaction had a total volume of 25 μL and contained 2.5 μL of target genomic DNA, corresponding to nominal target inputs of 50, 250, and 1, 250 copies per reaction.

For the no-matrix control, the standard RPA reaction mixture containing 4.1 μL of nuclease-free water was used. For the matrix-containing group, the 4.1 μL of nuclease-free water was replaced with an equal volume (4.1 μL) of genomic DNA extract from the negative vaginal specimen. All other RPA components and reaction conditions were identical between the two groups. Following RPA, the products were analyzed using the standard CRISPR/Cas12a and droplet-partitioned detection procedures described above. Each condition was tested using three separately prepared technical replicate reactions. Each reaction was independently emulsified, and three microscopic fields were analyzed per emulsion. The field-level positive-droplet counts were averaged to obtain one reaction-level value.

### 2.9 Clinical sample analysis

De-identified residual vaginal swab specimens originally obtained during routine clinical care were provided by the Second Affiliated Hospital of Jiaxing University (Zhejiang, China). The investigators did not directly recruit participants or collect specimens specifically for the present study. Routine clinical classification was established independently of the PddRPACas results using a composite assessment according to hospital practice that integrated clinical symptoms, microscopic examination, and a multiplex colloidal gold-based antigen assay for *Candida* spp., *Trichomonas vaginalis*, and *Gardnerella vaginalis*. The Candida component of the antigen assay detects *C. albicans*, *C. glabrata*, *C. parapsilosis*, and *C. tropicalis* and therefore does not provide species-level identification of *C. albicans*. A total of 28 specimens were included, comprising 18 composite-reference-positive and 10 composite-reference-negative specimens. The composite clinical reference classification and corresponding PddRPACas results for individual specimens are summarized in **Supplementary Table S4**. After collection during routine clinical care, residual vaginal swab specimens were stored at −20 °C until batch processing. The swabs were resuspended/diluted in the routine sample diluent, and genomic DNA was extracted using a commercial yeast genomic DNA extraction kit (Tiangen Biotech, Beijing, China) according to the manufacturer’s instructions. DNA was finally eluted in 200 μL. DNA concentration was not measured before PddRPACas testing; 2.5 μL of the DNA eluate was directly used as template in each 25 μL RPA reaction. The resulting RPA products were subsequently analyzed using the standard CRISPR/Cas12a and droplet-partitioned procedures described above. Because the specimens were de-identified residual samples, the exact collection dates and collection-to-extraction intervals were not available in the analytical dataset.

### 2.10 Analytical limit-of-detection verification

The analytical LoD was re-evaluated using target concentrations surrounding the previously estimated detection boundaries. For the bulk RPA-Cas12a assay, four separately prepared technical replicate reactions were tested at each concentration. Endpoint fluorescence at 45 min was used for classification, and the analytical positivity threshold was defined as the mean endpoint fluorescence of the NTC reactions plus three standard deviations. A reaction was classified as positive when its endpoint fluorescence exceeded this threshold. The operational LoD was defined as the lowest concentration at which all four technical replicate reactions were positive.

For the droplet-partitioned assay, three separately prepared technical replicate reactions were tested at each concentration. Each reaction was independently emulsified, and three microscopic fields were randomly acquired from each emulsion. Field-level positive-droplet counts were averaged to generate one reaction-level value. The positivity threshold was defined as the mean reaction-level positive-droplet count of the NTC plus three standard deviations. A reaction was classified as positive when its reaction-level value exceeded this threshold. The operational LoD was defined as the lowest concentration at which all three technical replicate reactions were positive.

### 2.11 Image processing and statistical analysis

Fluorescence images were analyzed using ImageJ version 1.54. Raw images were converted to 8-bit grayscale images. A fluorescence-intensity threshold defined from the no-template control (NTC) was applied consistently to images acquired under the same conditions. After binarization, the Fill Holes and Watershed functions were used to process and separate touching fluorescent objects, followed by the Analyze Particles function for positive-droplet counting. The same image-processing parameters were applied throughout each experiment. Unless otherwise specified, droplet-based analytical experiments were performed using three separately prepared technical replicate reactions per condition. Each reaction was independently emulsified, and three random microscopic fields were analyzed per emulsion. Field-level positive-droplet counts were averaged to generate one reaction-level value; individual microscopic fields were not treated as independent replicates. The bulk LoD verification used four separately prepared technical replicate reactions per concentration. Clinical specimens obtained from different individuals were treated as independent clinical samples. Analytical LoD was not determined by hypothesis testing but by predefined NTC-based positivity criteria. Exact binomial 95% confidence intervals were calculated for PPA, NPA, and overall clinical agreement. GraphPad Prism version 10.1 was used for data visualization and statistical calculations.

## 3 Results and discussion

### 3.1 Principle of the PddRPACas assay

The principle of the PddRPACas assay is shown in **Scheme 1**. In this strategy, target *Candida albicans* DNA is first amplified by RPA. The amplification products are then introduced into a Cas12a/crRNA/reporter reaction system and emulsified with oil by vortex mixing to generate polydisperse droplets. In droplets containing the target amplicon, sequence-specific recognition activates the trans-cleavage activity of Cas12a, resulting in cleavage of the fluorescent reporter and generation of localized fluorescence signals. In contrast, droplets lacking the target remain non-fluorescent. The fluorescence-positive droplets can then be visualized under a fluorescence microscope.

**Scheme 1:**
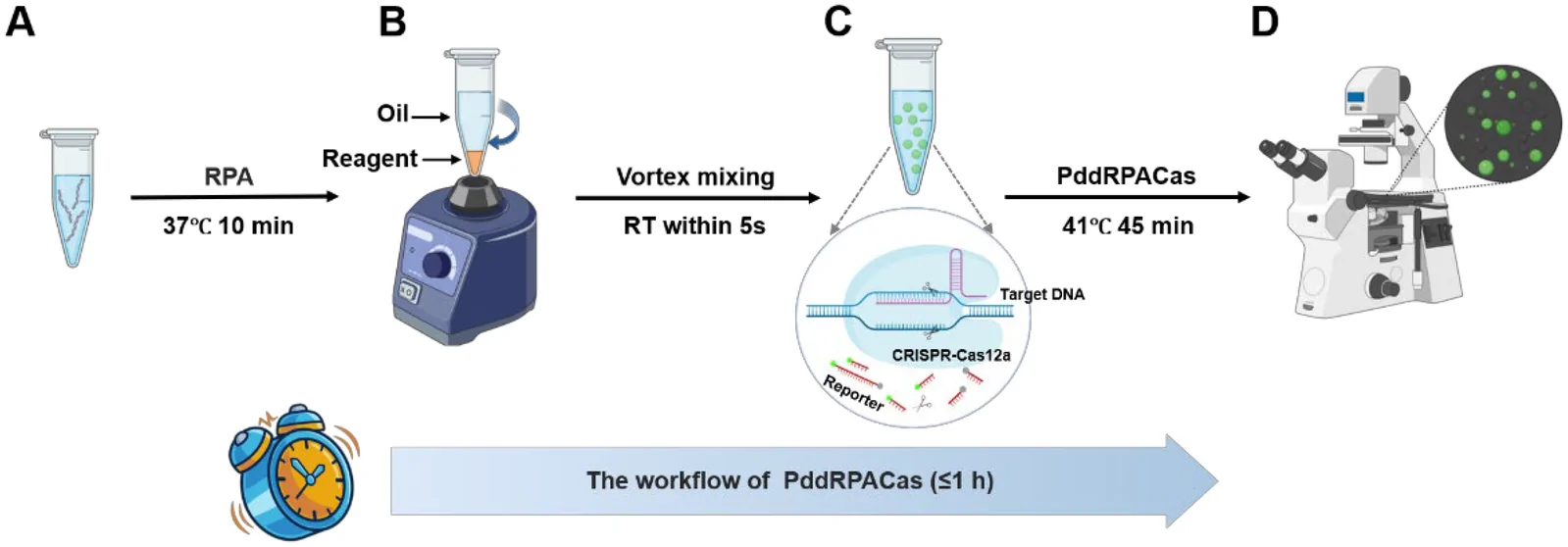
Schematic illustration of the PddRPACas assay for rapid detection of *Candida albicans* DNA. **(A)** Target DNA is first amplified by RPA at 37 °C for 10 min. **(B)** The amplified products are mixed with the CRISPR/Cas12a reaction system and oil phase, followed by vortexing to generate polydisperse droplets. **(C)** Within droplets, target-dependent Cas12a activation triggers trans-cleavage of the FAM-labeled ssDNA reporter and generates localized fluorescence signals during incubation at 41 °C for 45 min. **(D)** Fluorescence-positive droplets are visualized under a fluorescence microscope for droplet-partitioned fluorescence readout. The overall workflow can be completed within approximately 1 h.

Quantitative characterization of the vortex-generated emulsion showed a broad droplet-size distribution, with diameters ranging from approximately 10 to 50 μm (**Supplementary Figure S1**). More than 90% of the analyzed droplets were smaller than 30 μm, confirming the formation of predominantly small, polydisperse reaction compartments for the subsequent fluorescence readout. Therefore, the present assay is more appropriately interpreted as a droplet-partitioned fluorescence readout rather than a strict Poisson-based absolute quantification platform. In this context, compartmentalization primarily improves signal discrimination by spatially confining Cas12a activation events and reducing background interference. This interpretation is consistent with previous droplet Cas12a studies and supports a qualitative rather than absolute quantitative interpretation of the partitioned readout (Xu et al., 2025).

### 3.2 Feasibility of the RPA-assisted Cas12a detection system

To assess assay feasibility, we first established the overall experimental workflow for RPA-assisted bulk and droplet-partitioned Cas12a detection, as summarized in **Figure 1A**. The feasibility evaluation included upstream RPA amplification of the target sequence, Cas12a/crRNA-mediated reporter cleavage, and fluorescence readout in either bulk solution or droplet format. We then screened RPA primer pairs targeting the ITS region of *Candida albicans*. Multiple primer combinations produced the expected amplification bands, indicating successful target amplification. Among them, the F1/R4 primer pair produced the strongest band and was therefore selected for subsequent experiments. These results confirmed the feasibility of sequence-specific RPA amplification for the target region (**Supplementary Figure S2**).

**Figure 1:**
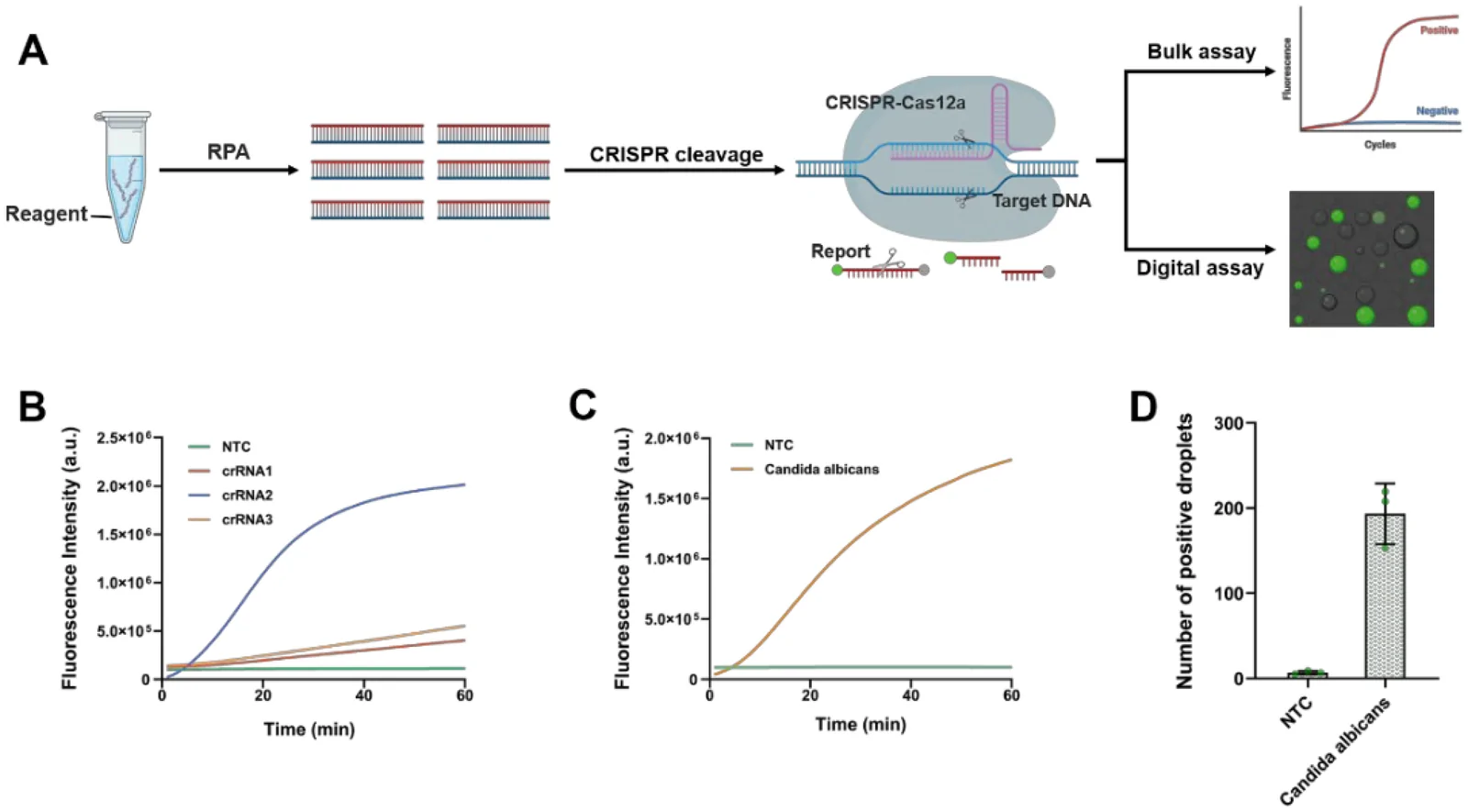
Feasibility of the RPA-assisted Cas12a detection system and droplet-partitioned PddRPACas readout for *Candida albicans* DNA detection. **(A)** Schematic workflow of the feasibility evaluation, including upstream RPA amplification, downstream Cas12a-mediated reporter cleavage, and bulk or droplet-based fluorescence readout. **(B)** Real-time fluorescence curves of Cas12a activation mediated by three candidate crRNAs targeting the ITS1/2 region of *Candida albicans*. NTC, no-template control. **(C)** Real-time fluorescence curves of the bulk RPA-Cas12a assay for target plasmid detection. NTC, no-template control. **(D)** Number of fluorescence-positive droplets detected by the PddRPACas assay in the target and NTC groups, demonstrating feasibility of the droplet-partitioned readout. NTC, no-template control. Data are represented as mean ± standard deviation with three technical replicates.

Next, three candidate crRNAs targeting the ITS1/2 region were evaluated in bulk Cas12a fluorescence assays. All crRNA-containing groups produced detectable fluorescence signals in the presence of *Candida albicans* genomic DNA, whereas the no-crRNA control remained negative. Among them, crRNA2 showed the strongest fluorescence response and the shortest time to reach plateau and was therefore selected for subsequent experiments (**Figure 1B**). The target-dependent activation of Cas12a was further confirmed using plasmid DNA containing the *Candida albicans* target sequence, where the target group produced a pronounced fluorescence increase compared with the no-template control (**Figure 1C**).

Among the three candidate guides, crRNA2 generated the strongest fluorescence response and reached the signal plateau most rapidly under the RPA-assisted reaction conditions. This observation indicates that crRNA performance should be evaluated within the complete analytical workflow rather than inferred solely from sequence design or predicted RNA folding. In the present assay, Cas12a recognition occurs in the context of amplified target products, and guide activity may therefore be influenced by amplicon abundance, local target accessibility, and the structural presentation of the target region. On the basis of the experimental screening results, crRNA2 was selected for all subsequent assays.

We then transferred the optimized target recognition system into the droplet-partitioned format. Fluorescence-positive droplets were automatically enumerated using ImageJ (**Supplementary Figure S3**). Compared with the no-template control, the *Candida albicans* group produced a markedly higher number of fluorescence-positive droplets, demonstrating the feasibility of the PddRPACas readout (**Figure 1D**). Representative droplet images are shown in **Supplementary Figure S4**.

### 3.3 Optimization of the PddRPACas assay

We next optimized the key parameters affecting assay performance. For the RPA step, reaction temperature, primer concentration, and Mg^2+^ concentration were systematically screened by gel electrophoresis (**Figure 2A**). Before optimization, the RPA reaction required approximately 30 min to approach saturation (**Figure 2B**), whereas after optimization effective amplification was achieved within approximately 10 min (**Figure 2C**). These results demonstrate that reaction optimization substantially improved amplification efficiency and reduced the amplification time.

**Figure 2:**
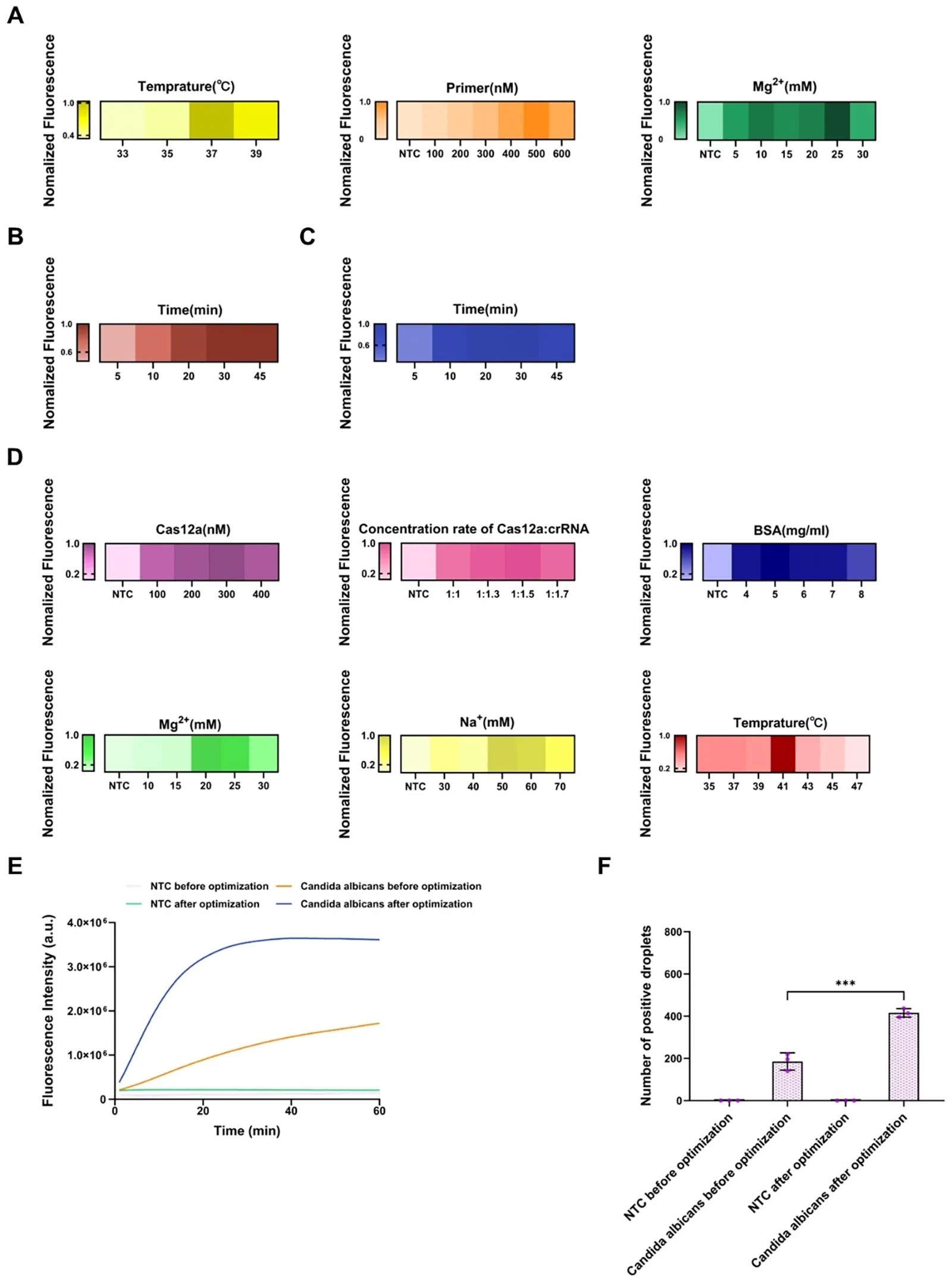
Optimization of the PddRPACas system. **(A)** Heatmap summary of RPA optimization, including reaction temperature, primer concentration, and Mg^2+^ concentration. Darker color indicates better amplification performance. **(B)** Heatmap summary of RPA amplification efficiency over time before optimization. **(C)** Heatmap summary of RPA amplification efficiency over time after optimization. **(D)** Heatmap-based optimization of the CRISPR/Cas12a reaction system, including Cas12a concentration, crRNA: Cas12a ratio, BSA concentration, Mg^2+^ concentration, Na^+^ concentration, and reaction temperature. Darker color indicates better performance. **(E)** Real-time fluorescence curves of the bulk RPA-Cas12a assay before and after optimization. NTC, no-template control. **(F)** Number of fluorescence-positive droplets before and after optimization, showing improved performance of the droplet-partitioned assay after coordinated optimization of the RPA and Cas12a steps. ***p < 0.001. Data are represented as mean ± standard deviation with three technical replicates.

For the Cas12a step, reaction temperature, Mg^2+^ concentration, BSA concentration, Cas12a concentration, and crRNA: Cas12a ratio were optimized by real-time fluorescence analysis (**Figure 2D**). The optimized conditions yielded a stronger and faster fluorescence response than the initial conditions, indicating improved target-triggered Cas12a activation (**Figure 2E**). When these optimized conditions were transferred to the droplet format, a larger number of fluorescence-positive droplets was observed, further confirming the benefit of coordinated optimization at both the amplification and readout levels (**Figure 2F**). Representative droplet images before and after optimization are shown in **Supplementary Figure S5**.

### 3.4 Analytical performance of the PddRPACas assay

To evaluate analytical sensitivity, plasmid DNA containing the target sequence was serially diluted and tested under the optimized conditions. Analytical detection boundaries were further verified using technical replicate reactions and predefined NTC-based positivity thresholds. For the bulk RPA-Cas12a fluorescence assay, four separately prepared technical replicate reactions were analyzed at each target concentration. A reaction was classified as positive when its endpoint fluorescence exceeded the mean endpoint fluorescence of the NTC reactions plus three standard deviations. Under this criterion, 10 copies/μL was the lowest target concentration detected in all four technical replicate reactions, whereas the lower tested concentrations were not consistently positive across all replicates. Thus, the operational LoD of the bulk assay was 10 copies/μL under the present experimental conditions (**Figure 3A**). Time-course analysis showed that positive droplets could be distinguished from the NTC after approximately 15 min and that the positive-droplet signal approached a maximum at approximately 45 min (**Supplementary Figure S6**). For the droplet-partitioned assay, three separately prepared technical replicate reactions were analyzed at each concentration. Each reaction was independently emulsified, and three microscopic fields were analyzed from each emulsion. The field-level positive-droplet counts were averaged to generate one reaction-level value. A reaction was classified as positive when this value exceeded the mean NTC reaction-level positive-droplet count plus three standard deviations. Under this criterion, 1 copy/μL was the lowest concentration detected in all three technical replicate reactions, whereas the lower tested concentrations were not consistently positive across all replicates (**Figure 3B**; **Supplementary Figure S7**).

**Figure 3:**
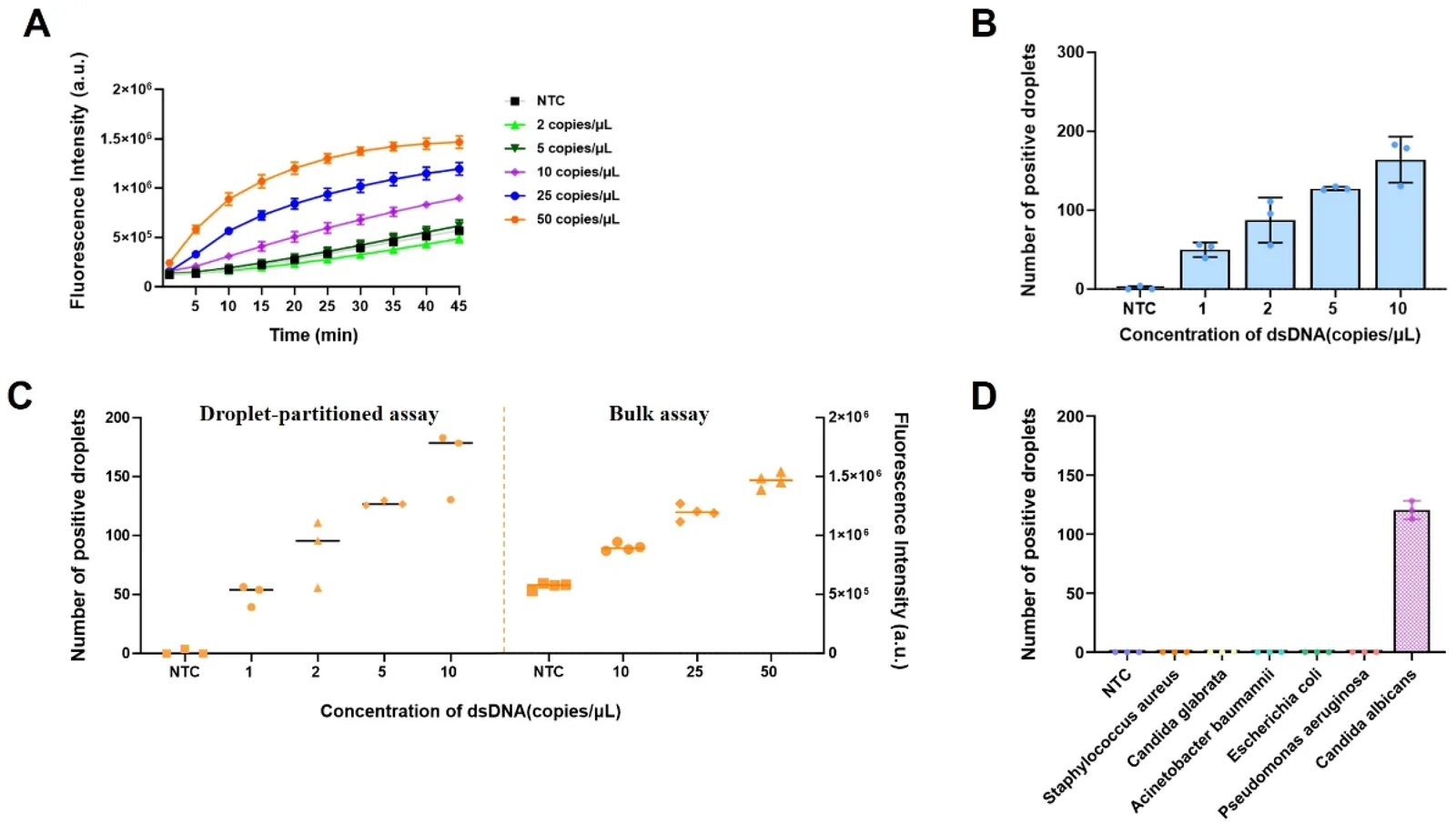
Analytical performance and specificity of the PddRPACas assay for *C. albicans* DNA detection. **(A)** Bulk RPA-Cas12a fluorescence responses at different pre-RPA target concentrations. Four technical replicate reactions were analyzed per concentration; the analytical positivity threshold was defined as the mean endpoint fluorescence of the NTC plus three standard deviations. Data are represented as mean ± standard deviation with four technical replicates. **(B)** Reaction-level positive-droplet counts obtained with PddRPACas at different pre-RPA target concentrations. Data are represented as mean ± standard deviation with three technical replicates. **(C)** Side-by-side comparison of reaction-level responses across target concentrations for the droplet-partitioned and bulk fluorescence formats. **(D)** Analytical specificity of the PddRPACas assay against the tested target and non-target organisms. Data are represented as mean ± standard deviation with three technical replicates.

Because 2.5 μL of template was added to each 25 μL RPA reaction, pre-RPA concentrations of 10 and 1 copy/μL corresponded to nominal inputs of 25 and 2.5 copies per RPA reaction, respectively. Subsequently, 1.25 μL of RPA product was transferred into each 12.5 μL Cas12a reaction. Target concentrations reported here therefore refer to the pre-RPA template concentrations rather than the post-amplification copy numbers. Direct comparison of the two readout formats showed that the lowest concentration consistently detected across all tested technical replicates decreased from 10 copies/μL in the bulk fluorescence format to 1 copy/μL in the droplet-partitioned format, corresponding to an approximately 10-fold improvement in analytical detectability (**Figure 3C**). Because the number of replicate reactions was limited, these values are interpreted as operational LoDs under the present experimental conditions rather than formal LoD95 estimates. Because the vortex-generated droplets were polydisperse and were not subjected to Poisson-based analysis, the assay is interpreted as a qualitative droplet-partitioned readout rather than an absolute digital quantification method.

### 3.5 Analytical specificity and in-silico sequence evaluation

To evaluate analytical specificity, genomic DNA from *C. albicans* was tested together with the original non-target panel and additional clinically relevant non-*albicans Candida* species. The expanded panel included *C. dubliniensis*, *C. glabrata, C. tropicalis, C. parapsilosis, C. krusei*, and *C. auris*, as well as *P. aeruginosa, S. aureus, A. baumannii*, and *E. coli* (**Supplementary Table S3**). Under the same assay conditions, the *C. albicans* group generated clear fluorescence-positive droplet signals, whereas none of the tested non-target organisms exceeded the analytical positivity threshold (**Figure 3D**; **Supplementary Figure S8**). *C. albicans* also remained detectable in the mixed-*Candida* background, supporting target recognition in the presence of additional non-target *Candida* DNA (**Supplementary Figures S8B, C**). Thus, no cross-reactivity was observed with the non-target organisms evaluated under the present experimental conditions. To further assess the sequence basis of assay specificity, the target region was compared with homologous ITS-region sequences from *C. auris, C. dubliniensis, C. parapsilosis, C. tropicalis*, and *C. krusei*. Multiple nucleotide mismatches were observed within the RPA primer-binding and/or crRNA2-binding regions of the non-target species, and the adjacent TTTC PAM was not conserved in several of these sequences (**Supplementary Figures S9A, B**). These sequence differences are expected to reduce efficient RPA amplification and/or Cas12a target recognition and provide a sequence-based rationale for the experimentally observed lack of cross-reactivity with the tested non-target *Candida* species.

We additionally evaluated sequence conservation of the assay-binding regions within *C. albicans*. Alignment of ITS-region sequences from 11 *C. albicans* clinical isolates (**Supplementary Table S2**) showed no nucleotide substitutions within the F1 primer-binding site, the crRNA2 protospacer, or the R4 primer-binding site among the sequences analyzed (**Supplementary Figure S9C**). These findings support conservation of the selected primer- and crRNA-binding sites within the *C. albicans* isolates included in the in-silico analysis. Together, the experimental specificity results and in-silico sequence analyses support the selectivity of the current primer/crRNA combination for *C. albicans* among the organisms evaluated. Nevertheless, these findings should not be interpreted as establishing universal exclusivity against all possible fungal strains or sequence variants.

### 3.6 Effect of extracted vaginal DNA matrix on PddRPACas detection

To evaluate whether components remaining in DNA extracts from vaginal specimens interfere with the PddRPACas workflow, *C. albicans* genomic DNA at pre-RPA concentrations of 20, 100, and 500 copies/μL was tested in the absence or presence of DNA extract from a composite-reference-negative and qPCR-negative vaginal specimen (**Supplementary Figure S10**). The extracted vaginal DNA matrix was introduced during the RPA step by replacing the 4.1 μL of nuclease-free water in the standard 25 μL RPA reaction with an equal volume of the clinical DNA extract. *C. albicans* target DNA was successfully detected at all three concentrations in the presence of the extracted vaginal DNA matrix. At each target concentration, the matrix-containing reactions showed modestly lower positive-droplet counts than the corresponding no-matrix controls. Nevertheless, a clear concentration-dependent increase in positive-droplet counts was maintained, with progressively greater signals observed at 20, 100, and 500 copies/μL. These results indicate that the tested extracted vaginal DNA matrix exerted a modest inhibitory effect on the RPA-to-Cas12a droplet-detection workflow but did not prevent target detection under the conditions evaluated. Because the matrix was obtained after DNA extraction and only one negative clinical matrix was examined, these findings should be interpreted as an assessment of post-extraction matrix interference, rather than DNA-extraction recovery or comprehensive validation across the biological diversity of vaginal specimens.

### 3.7 Preliminary clinical concordance evaluation using vaginal swab specimens

To preliminarily evaluate clinical concordance, we analyzed 28 de-identified residual vaginal swab specimens, including 18 composite-reference-positive and 10 composite-reference-negative specimens according to the routine clinical reference described in Section 2.9 (**Figure 4A**). An exploratory classification cutoff of 53 positive droplets was derived from the 10 composite-reference-negative specimens as the mean positive-droplet count plus three standard deviations (mean = 17.8; SD = 11.5). Using this cohort-derived cutoff, all 18 composite-reference-positive and all 10 composite-reference-negative specimens were concordantly classified by PddRPACas (**Figures 4B, C**; **Supplementary Table S4**). Relative to the composite clinical reference, the positive percent agreement (PPA) was 100% (18/18; 95% CI, 81.5–100%), the negative percent agreement (NPA) was 100% (10/10; 95% CI, 69.2–100%), and the overall agreement was 100% (28/28; 95% CI, 87.7–100%). Because the composite clinical reference was not species-specific for *C. albicans*, these results should be interpreted as preliminary concordance rather than definitive diagnostic sensitivity or specificity.

**Figure 4:**
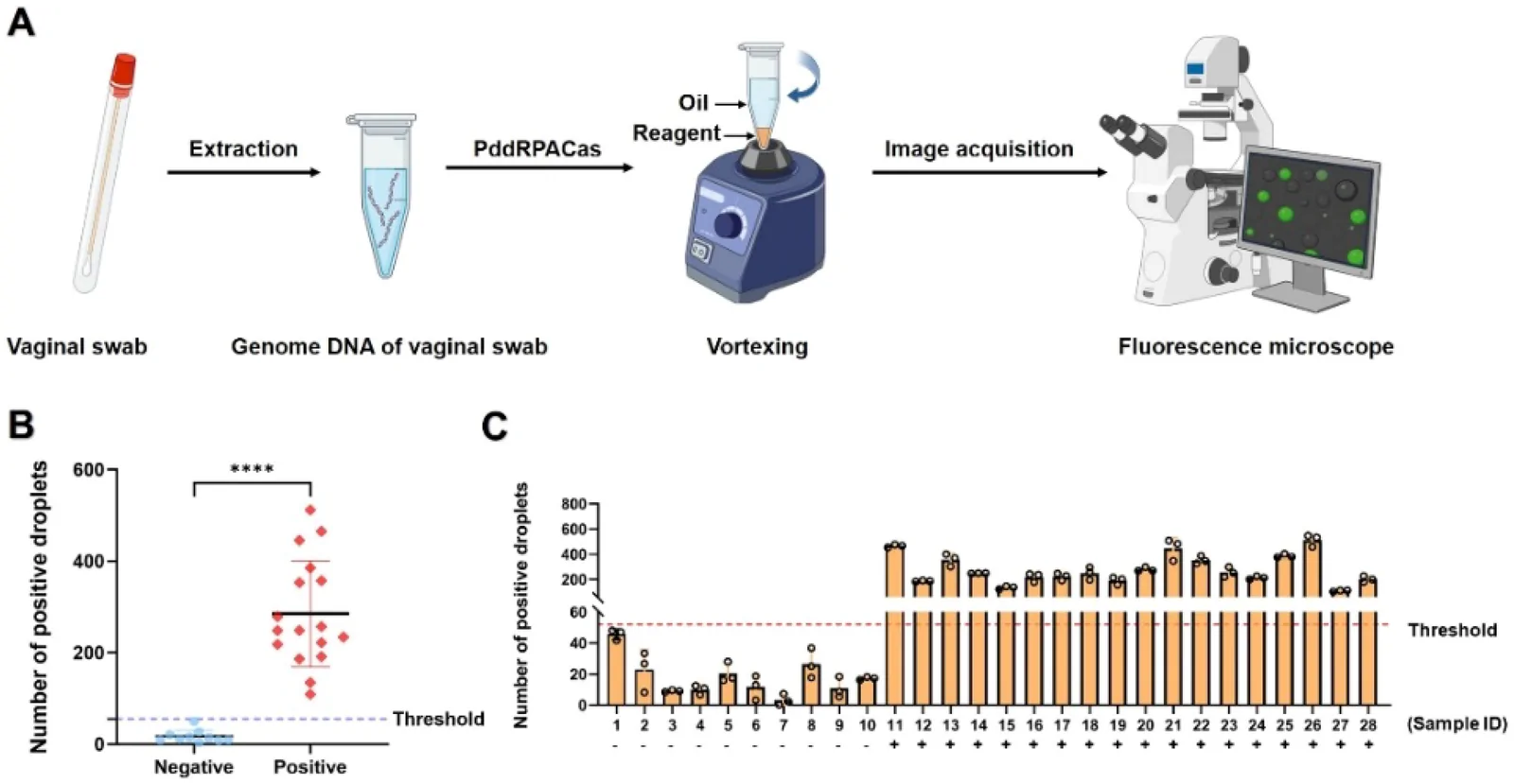
Preliminary clinical evaluation of the PddRPACas assay using vaginal specimens. **(A)** Schematic workflow for clinical sample analysis, including genomic DNA extraction from vaginal specimens, PddRPACas reaction, vortex-assisted droplet generation, and fluorescence imaging. **(B)** Scatter plot of fluorescence-positive droplet counts in composite-reference-negative and composite-reference-positive specimens; the dashed line indicates the exploratory cohort-derived cutoff of 53 positive droplets. ****p < 0.0001. **(C)** Bar chart summary of PddRPACas results for the clinical cohort, showing sample-to-sample variation in droplet-positive signals across the tested specimens. Data are represented as mean ± standard deviation with three technical replicates.

Although some variation in droplet counts was observed among clinical samples, such variation is not unexpected and may reflect differences in sample storage, DNA extraction efficiency, target burden, or matrix-associated interference. Importantly, despite these sources of variability, the assay maintained correct qualitative classification of all tested samples, supporting the feasibility of the assay for preliminary *C. albicans* DNA detection in vaginal specimens.

Using the exploratory cohort-derived cutoff of 53 positive droplets, all 18 composite-reference-positive specimens and all 10 composite-reference-negative specimens were concordantly classified. The resulting PPA, NPA, and overall agreement with the composite clinical reference were 100% within the present cohort. These values should not be interpreted as species-specific diagnostic sensitivity or specificity because the clinical comparator was not specific for *C. albicans*. The clinical results should nevertheless be interpreted as preliminary. The positivity cutoff was derived from the negative specimens included in the same cohort, and an independent validation set was not available. In addition, variation in positive droplet counts may reflect differences in fungal burden, specimen storage, DNA extraction efficiency, and matrix-associated inhibition. Larger independent and blinded cohorts will therefore be required to establish robust estimates of diagnostic performance and to validate the clinical classification threshold. The current workflow also involves transferring RPA products into the Cas12a reaction before droplet generation, which creates a potential risk of amplicon contamination. Future development should focus on closed-tube integration, contamination-control strategies, and simplified sample preparation before the assay can be considered for routine clinical implementation. In addition, the additional matrix-interference experiment demonstrated that target detection was retained when a representative extracted vaginal DNA matrix was present during RPA, although positive-droplet counts were modestly reduced. However, only one reference-negative clinical DNA matrix was evaluated, and the experiment did not include target spiking before DNA extraction. Therefore, the present data do not establish extraction recovery or matrix robustness across the heterogeneity of clinical vaginal specimens. Moreover, the present droplet repeatability assessment was based on separately prepared technical replicate reactions performed within the same experimental run. Although these data support within-run technical repeatability, broader inter-day, inter-operator, reagent-lot, and inter-laboratory reproducibility remains to be established. Importantly, PddRPACas detects *C. albicans* DNA and cannot distinguish symptomatic infection from asymptomatic colonization. Therefore, a positive result should be interpreted together with clinical manifestations and other microbiological findings rather than being considered independently diagnostic of VVC.

## 4 Conclusion

In this study, we established an RPA-assisted droplet-partitioned CRISPR/Cas12a assay for low-copy detection of *C. albicans* DNA. Using predefined NTC-based analytical positivity criteria, the operational LoD was 10 copies/μL for the bulk RPA-Cas12a format and 1 copy/μL for the droplet-partitioned format under the tested conditions. No cross-reactivity was observed with the non-target organisms evaluated, including additional clinically relevant non-*albicans Candida* species.

Preliminary testing of 28 residual vaginal specimens showed complete agreement with the routine composite clinical reference within the present cohort. However, this comparator was not species-specific for *C. albicans*, the clinical cutoff was derived from the same small cohort, and no independent validation population was available. In addition, detection of *C. albicans* DNA cannot distinguish active VVC from asymptomatic colonization. The present clinical findings should therefore be interpreted as preliminary concordance data. Larger independent studies using species-specific reference methods, together with broader reproducibility testing, closed-tube integration, and further matrix evaluation, will be required before clinical diagnostic performance can be established.

## Data Availability

The original contributions presented in the study are included in the article/**Supplementary Material**. Further inquiries can be directed to the corresponding authors.
