# Supplementary material for: RPA-primed droplet-partitioned CRISPR/Cas12a assay for low-copy detection of Candida albicans in vaginal specimens
Source: Front Cell Infect Microbiol. 2026 Oct 28;16:1960866. doi: 10.3389/fcimb.2026.1960866 (PMC13751328; doi:10.3389/fcimb.2026.1960866)
Supplement: Supplementary file 1 [file SupplementaryFile1.docx]

Supporting Information

Supplementary Figures

Figure S1. Representative image (A) and size distribution analysis (B) of polydisperse droplets generated by vortex emulsification. Droplet diameters were quantified from microscopy images to characterize the size distribution of the vortex-generated droplets. A total of n = 1000 droplets from microscopic fields were measured for the size-distribution analysis.

Figure S2. Agarose gel electrophoresis analysis of RPA amplicons generated using different primer pairs for *Candida albicans* detection. NTC, no-template control (water as template).

Figure S3. Data processing workflow using ImageJ to count positive droplets for (A) NTC and (B) positive control. Further information can be found in the “Data Processing and Statistical Analysis” section.

Figure S4. Representative droplet fluorescence images of the PddRPACas assay for *Candida albicans* DNA detection, corresponding to Fig. 1D. NTC, no-template control (water as template).

Figure S5. Representative droplet fluorescence images of the PddRPACas assay for *Candida albicans* DNA detection before and after optimization, corresponding to Fig. 2F. NTC, no-template control (water as template).

Figure S6. Representative droplet fluorescence images (A) and corresponding quantification of positive droplet counts (B) for the time-course evaluation of the PddRPACas assay at different incubation times, corresponding to the time-dependent analysis described in the main text. Data are represented as mean ± standard deviation with three technical replicates.

Figure S7. Quantification of positive droplet counts in the PddRPACas assay using serially diluted plasmid DNA containing the *Candida albicans* target sequence at different concentrations, corresponding to Fig. 3B. NTC, no-template control (water as template).

Figure S8. Specificity assessment of the PddRPACas assay. (A) Representative fluorescence images for common non-target microorganisms, corresponding to Fig. 3D. (B) Representative fluorescence images for other clinically relevant *Candida* species and mixed *Candida* spp. (C) Quantification of positive droplet counts corresponding to (B). *Candida albicans* serves as the positive control. NTC, no-template control (water as template). Mixed Candida spp. represents a mixed-background sample containing *C. albicans* together with *C. auris*, *C. dubliniensis*, *C. parapsilosis*, *C. tropicalis*, and *C. krusei*. Data are represented as mean ± standard deviation with three technical replicates.

Figure S9. In-silico sequence analysis of the PddRPACas target region. (A) Multiple-sequence alignment of the target region among *C. albicans* and clinically relevant non-*albicans Candida* species. The RPA primer-binding regions are indicated by blue arrows, the crRNA2 protospacer is highlighted in yellow, and the adjacent TTTC PAM is indicated in red. (B) Enlarged schematic of the crRNA2 protospacer and adjacent PAM. (C) Multiple-sequence alignment of the target region from 11 *C. albicans* clinical isolate sequences. The F1 primer-binding region (5’-CCACATGTGTTTTTCTTTGAAACAAACTTGC-3’), crRNA2 protospacer (5’-TTTGAAACAAACTTGCTTTG-3’), and R4 primer-binding region (5’-CTGCAATTCATATTACGTATCGCATTTCGCTGC-3’) are indicated. No nucleotide substitutions were observed within these assay-binding regions among the sequences analyzed.

Figure S10. Effect of extracted vaginal DNA matrix on PddRPACas detection. (A) Representative droplet fluorescence images. (B) Statistics of positive droplet counts for samples with different target concentrations in the presence and absence of extracted vaginal DNA matrix. The target concentrations corresponding to weak positive, moderate positive, and strong positive are 20, 100, and 500 copies/μL, respectively. Data are represented as mean ± standard deviation with three technical replicates.

Supplementary Tables

Table S1. Oligonucleotide sequences used in this study.

| Name | Sequence (5′→3′) |
| --- | --- |
| *Candida albicans* target sequence | GATCATTACTGATTTGCTTAATTGCACCACATGTGTTTTTCTTTGAAACAAACTTGCTTTGGCGGTGGGCCCAGCCTGCCGCCAGAGGTCTAAACTTACAACCAATTTTTTATCAACTTGTCACACCAGATTATTACTAATAGTCAAAACTTTCAACAACGGATCTCTTGGTTCTCGCATCGATGAAGAACGCAGCGAAATGCGATACGTAATATGAATTGCAGATATTCGTGAATCATCGAATCTTTGAACGCACATTGCGCCCTCTGGTATTCCGGAGGGCATGCCTGTTTGAGCGTCGTTTCTCCCTCAAACCGCTGGGTTTGGTGTTGAGCAATACGACTTGGGTTTGCTTGAAAGACGGTAGTGGTAAGGCGGGATCGCTTTGACAATGGCTTAGGTCTAACCAAAAACATTGCTTGCGGCGGTAACGTCCACCACGTATATCTTCAAACTTTGACCTCAAATCAGGTAGGACTACCCGCTGAACTTAAGCATATCAATAAGCGGAGGAAAAGAAA |
| ITS RPA F1 | CCACATGTGTTTTTCTTTGAAACAAACTTGC |
| ITS RPA F2 | CCCAGCCTGCCGCCAGAGGTCTAAACTTAC |
| ITS RPA F3 | CAAACTTGCTTTGGCGGTGGGCCCAGCCTGCCGC |
| ITS RPA F4 | CCTCTGGTATTCCGGAGGGCATGCCTGTTTGAGC |
| ITS RPA F5 | CTCCCTCAAACCGCTGGGTTTGGTGTTGAGC |
| ITS RPA R1 | TGTAAGTTTAGACCTCTGGCGGCAGGCTGGGC |
| ITS RPA R2 | CGTTCTTCATCGATGCGAGAACCAAGAGATCC |
| ITS RPA R3 | CGTATCGCATTTCGCTGCGTTCTTCATCGATGC |
| ITS RPA R4 | CTGCAATTCATATTACGTATCGCATTTCGCTGC |
| ITS RPA R5 | CCTAAGCCATTGTCAAAGCGATCCCGC |
| ITS RPA R6 | CAATGTTTTTGGTTAGACCTAAGCCATTGTCAAAGC |
| ITS RPA R7 | CCGCAAGCAATGTTTTTGGTTAGACCTAAGC |
| crRNA1  crRNA2  crRNA3  Reporter DNA | UAAUUUCUACUAAGUGUAGAUCUUGAAAGACGGUAGUGGUA  UAAUUUCUACUAAGUGUAGAUUUUGAAACAAACUUGCUUUG  UAAUUUCUACUAAGUGUAGAUUCAACUUGUCACACCAGAUU  6-FAM-TTTCTGTCATTC-BHQ1 |

Note: Yellow shading indicates the target region recognized by crRNA1; gray shading indicates the target region recognized by crRNA2; red shading indicates the target region recognized by crRNA3. Blue letters indicate the spacer sequence of each crRNA.

Table S2. NCBI accession information for 11 Candida albicans clinical isolate sequences used for in-silico conservation analysis.

| Number | Accession | Species | Source |
| --- | --- | --- | --- |
| 1 | MN336231.1 | *Candida albicans* | Vulvovaginal Candidiasis |
| 2 | MK138363.1 | *Candida albicans* | vaginal swab |
| 3 | MH084789.1 | *Candida albicans* | human oral swab |
| 4 | PZ556049.1 | *Candida albicans* | respiratory secretions |
| 5 | KU052044.1 | *Candida albicans* | blood |
| 6 | KC408953.1 | *Candida albicans* | Clinical isolate |
| 7 | PZ723410.1 | *Candida albicans* | Clinical-CMCC |
| 8 | KX355315.1 | *Candida albicans* | Homo sapiens; male |
| 9 | PV133306.1 | *Candida albicans* | urine |
| 10 | PQ613286.1 | *Candida albicans* | urine |
| 11 | KT585566.1 | *Candida albicans* | blood |

Table S3. *Candida* species used for analytical specificity evaluation and corresponding reference sequence accession numbers.

| Name | Accession |
| --- | --- |
| *Candida albicans* gDNA | OW987719.1 |
| *C. parapsilosis* gDNA | LT577616.1 |
| *C.tropicalis* gDNA | OW984071.1 |
| *C.dubliniensis* gDNA | OW985868.1 |
| *C.auris* gDNA | OW988727.1 |
| *C. krusei* gDNA | OZ376038.1 |
| *C. glabrata* gDNA | GCA_010111755.1 |

Table S4. Composite clinical reference classification and PddRPACas results for vaginal specimens included in this study.

| Sample ID | Age (years) | Composite clinical reference | PddRPACas result |
| --- | --- | --- | --- |
| 1 | NA | - | Negative |
| 2 | NA | - | Negative |
| 3 | 29 | - | Negative |
| 4 | 20 | - | Negative |
| 5 | 39 | - | Negative |
| 6 | 48 | - | Negative |
| 7 | 31 | - | Negative |
| 8 | 34 | - | Negative |
| 9 | 38 | - | Negative |
| 10 | 40 | - | Negative |
| 11 | 50 | + | Positive |
| 12 | 34 | + | Positive |
| 13 | NA | + | Positive |
| 14 | 48 | + | Positive |
| 15 | NA | + | Positive |
| 16 | 20 | + | Positive |
| 17 | 30 | + | Positive |
| 18 | 22 | + | Positive |
| 19 | 53 | + | Positive |
| 20 | 44 | + | Positive |
| 21 | 40 | + | Positive |
| 22 | 46 | + | Positive |
| 23 | NA | + | Positive |
| 24 | NA | + | Positive |
| 25 | 37 | + | Positive |
| 26 | 20 | + | Positive |
| 27 | 33 | + | Positive |
| 28 | 39 | + | Positive |

Note: NA, age unavailable; Symbols: +, positive composite clinical reference result; −, negative composite clinical reference result. “Positive” denotes a positive PddRPACas assay outcome, and “negative” denotes a negative PddRPACas assay outcome.
